# Ivermectin under scrutiny: a systematic review and meta-analysis of efficacy and possible sources of controversies in COVID-19 patients

**DOI:** 10.1186/s12985-022-01829-8

**Published:** 2022-06-13

**Authors:** Arman Shafiee, Mohammad Mobin Teymouri Athar, Omid Kohandel Gargari, Kyana Jafarabady, Sepehr Siahvoshi, Sayed-Hamidreza Mozhgani

**Affiliations:** 1 Student Research Committee, Alborz University of Medical Sciences, Karaj, Iran; 2 School of Medicine, Alborz University of Medical Sciences, Karaj, Iran; 3 School of Medicine, Shahid Beheshti University of Medical Sciences, Tehran, Iran; 4 Dental Materials Research Center, Dental School, Islamic Azad University of Medical Sciences, Tehran, Iran; 5 Department of Microbiology, School of Medicine, Alborz University of Medical Sciences, Karaj, Iran; 6 Non-Communicable Disease Research Center, Alborz University of Medical Sciences, Karaj, Iran

**Keywords:** COVID-19, SARS-CoV-2, Ivermectin, Treatment, Meta-analysis, Mortality

## Abstract

**Background:**

We conducted a systematic review and meta-analysis to evaluate the efficacy of ivermectin for COVID-19 patients based on current peer-reviewed RCTs and to address disputes over the existing evidence.

**Methods:**

MEDLINE (Pubmed), Scopus, Web of Science, Cochrane library, Google scholar and Clinicaltrials.gov were searched for RCTs assessing the efficacy of Ivermectin up to 20 February 2022. A systematic review and meta-analysis of studies was performed based on the PRISMA 2020 statement criteria.

**Results:**

19 and 17 studies were included in this systematic review and meta-analysis, respectively. There was no significant difference in progression to severe disease (log OR − 0.27 [95% CI − 0.61 to 0.08], I2 = 42.29%), negative RT-PCR (log OR 0.25 [95% CI − 0.18–0.68], I2 = 58.73%), recovery (log OR 0.11 [95% CI − 0.22–0.45], I2 = 13.84%), duration of hospitalization (SMD − 0.40 [95% CI − 0.85–0.06], I2 = 88.90%), time to negative RT-PCR (SMD − 0.36 [95% CI − 0.89–0.17], I2 = 46.2%), and viral load (SMD -0.17 [95% CI -0.45 to 0.12], I^2 = 0%). It is worth noting that, based on low-certainty evidence, ivermectin may possibly reduce mortality (log OR − 0.67 [95% CI − 1.20 to − 0.13], I2 = 28.96%). However, studies with a higher risk of bias were more likely to indicate positive effects on the efficacy of this drug, according to our subgroup analyses based on study quality.

**Conclusion:**

Ivermectin did not have any significant effect on outcomes of COVID-19 patients and as WHO recommends, use of ivermectin should be limited to clinical trials.

**Supplementary Information:**

The online version contains supplementary material available at 10.1186/s12985-022-01829-8.

## Introduction

In December 2019, a cluster of cases of pneumonia with unknown etiology was reported in Wuhan, China [1]. Patients presented to healthcare facilities with flu-like symptoms such as dyspnea, dry cough, and fever. In January 2020 the agent causing the disease was named severe acute respiratory syndrome-coronavirus 2 (SARS-CoV-2). This unprecedented situation left health providers with lack of sufficient information regarding the source and means of transmission of the virus resulting in the inability to prevent the rapid spread of the disease throughout the world. On 11th March 2020, novel coronavirus disease (COVID-19) caused by SARS-CoV-2 was declared a pandemic by WHO [2].

Since the emergence of novel coronavirus disease (COVID-19), over 5 million worldwide deaths have been reported by WHO [3]. Attempts were made to implement appropriate treatment strategies against the disease. Although there is no certain treatment approved by official health organizations for most patients in the early stage of the disease, pharmacotherapies including drugs such as hydroxychloroquine, azithromycin, and remdesivir have been employed as possible treatments for COVID-19 [4]. Several clinical trials were conducted to determine the efficacy of candidate drugs, however further investigations on other promising drugs are still required [5].

Ivermectin, a low-cost, simple-to-use, and widely available FDA-approved anti-parasitic drug, is one of the proposed drugs for treating COVID-19 patients [6], and it has sparked one of the greatest debates since the beginning of the pandemic [7–10]. An in-vitro study published by Caly et al.[11] reported that ivermectin inhibits SARS-CoV-2 replication with a 5000-fold reduction in viral RNA level during the first 48 h of usage. Although ivermectin is considered an anti-parasite agent, there is some evidence that proves its efficacy against viruses [12, 13]. Additionally, a preprint trial by Elgazzar et al. [] showed encouraging positive effects of ivermectin on COVID-19 patients. Following these publications, several low-quality studies were conducted to evaluate the effect of ivermectin on COVID-19 patients [14, 15]. These studies were included in a number of systematic reviews and meta-analyses that reported the beneficial effect of this medication [14, 16–18]. Furthermore, recent RCTs have shown that ivermectin is ineffective in the treatment of COVID-19 [19, 20]. Since the beginning of the pandemic, all of these have caused controversy regarding the possible effects of this drug. And despite the fact that WHO recommends that ivermectin must be used solely in clinical trials to treat COVID-19, according to recently published studies, the misuse of this medication and a significant number of ivermectin prescriptions have been widely documented, particularly in the United States [9, 10].

After about two years since the beginning of the COVID-19 pandemic, valuable lessons were achieved from ivermectin in the context of COVID-19 for investigating future proposed therapeutic targets in the era of the pandemic, which are of high importance for both researchers and clinicians. Moreover, further information regarding possible therapeutic agents that would reduce mortality and change the course of COVID-19 is desperately needed. There are some concerns regarding the accuracy of the results of previous studies that should be addressed. The most important concerns are several retractions of clinical trials [21], the availability of inaccurate or outdated meta-analyses [14, 22, 23], and three RCTs recently published in 2022 [19, 20, 24] which their final results were not included in previous meta-analyses. Considering these issues, we set out to conduct a comprehensive systematic review and meta-analysis of current peer-reviewed randomized controlled trials to assess the possible effect of ivermectin in patients with COVID-19. We also reviewed and discussed the possible sources of the controversial opinions regarding this drug, assessed the current state of available ivermectin systematic reviews and meta-analyses, and highlighted key elements that could lead to reliable investigations into potential therapeutic targets in future research.

## Methods

### Search strategy

We have performed a systematic review and meta-analysis in accordance with the recommendations from the Cochrane Handbook [25]. The Preferred Reporting Items for Systematic Reviews and Meta-Analyses (PRISMA) was used in this study [26]. The protocol of our study is registered at Alborz University of Medical Sciences with the number IR.ABZUMS.REC.1400.321.

MEDLINE (Pubmed), Scopus, Web of Science, Cochrane library, Google scholar and Clinicaltrials.gov were assessed by our reviewers (A.S and M.T) who designed a search strategy using the search string: (((((("COVID-19"[Mesh]) OR ("SARS-CoV-2"[Mesh])) OR (COVID-19)) OR (Coronavirus)) OR (nCoV)) OR (SARS-Cov-2)) AND ((((((("Ivermectin"[Mesh]) OR (MK-933)) OR (Stromectol)) OR (Mectizan)) OR (Eqvalan)) OR (Ivomec)) OR (Ivermectin)) (Further details available in Additional file 1: Table S1 supplementary data). All publications were retrieved up to 20 February 2022. We also investigated the reference sections of other systematic reviews and meta-analyses for relevant RCTs. Pre-print databases were not included due to concerns around non-peer-reviewed studies regarding ivermectin [14, 15]. The result was exported to the EndNote X9 program for further screening.

### Study selection and data extraction

Articles eligible for inclusion were: 1) confirmed COVID-19 patients, 2) randomized clinical trials comparing ivermectin to standard of care (SOC) or placebo, 3) evaluated relevant outcomes in this topic. To avoid low-quality studies affecting the overall results, we excluded non-randomized trials, observational studies, and non-peer-reviewed databases. Screening was performed in duplicate and two-step selection has been undertaken by two reviewers (A.S and M.T). Studies were screened via titles and abstracts followed by full-texts in EndNote. All disagreements were resolved by means of discussion with a third reviewer to reach an agreement (K.J). Data were extracted from text, tables, figures, graphs, and supplementary materials. Author name, year of publication, country of origin, study design, intervention and control arm descriptions, total number of patients in each trial, and outcome data were all extracted by three reviewers independently (A.S and M.T and S.S). In case of studies with unavailable or inadequate data, we attempted to contact the corresponding author to receive the unpublished data. Studies reporting median and interquartile range for their outcomes were analyzed for no significant skewness [27]. The estimated mean and standard deviation of eligible studies were used for meta-analysis [28, 29].

### Quality assessment

Two reviewers (K.J and M.T) independently assessed the included studies using Cochrane ROB-2 tool [30]. All included studies were rated as “High”, “Some concerns”, and “Low” based on ROB-2 checklist. We assessed the quality of evidence for our outcomes using the Grading of Recommendations, Assessment, Development and Evaluations (GRADE) framework [31].

### Outcome measure

Our primary outcomes include rate of mortality, progression to severe or critical state, and negative RT-PCR. Our secondary outcomes include recovery rate, duration of hospitalization, time to negative RT-PCR, and viral load.

### Data synthesis and analysis

Data was pooled using the random-effects method because the indicators were supposed to vary across studies and there was variability among the studies. The log odds ratio (log OR) was used to summarize the overall effect of dichotomous outcomes and the standardized mean difference (SMD) was used to describe the overall effect of continuous outcomes. Study heterogeneity was assessed using I2 statistic, with I2 values of < 50%, 50% to 75%, and > 75% indicating low, moderate, and high heterogeneity, respectively. While I2 is the most commonly used measure of heterogeneity, the I2 measure increases with an increasing number of trials, making it harder to compare I2 across analyses, therefore we report both I2 and Tau for each analysis under primary outcomes. Publication bias was assessed using funnel plots and Egger’s regression test for funnel plot asymmetry. In terms of publication bias existence, a trim and fill analysis were used to evaluate the number of missing studies and the effect of imported studies on effect size. Subgroup analysis were performed to seek the possible effect of study quality and funding on overall effect. Sensitivity analysis was conducted by using leave-one-out analysis to assess the individual study effect on pooled results. A meta-regression was used in terms of moderate or high heterogeneity to investigate the association between effect size and covariates. Publication bias, subgroup, sensitivity, and meta-regression analyses were only done on primary outcomes as a small number of studies have been included in the secondary outcomes. Stata version 17 statistical software (Stata Corp, College Station, TX, USA) was used for quantitative synthesis.

## Results

**Fig. 1:**
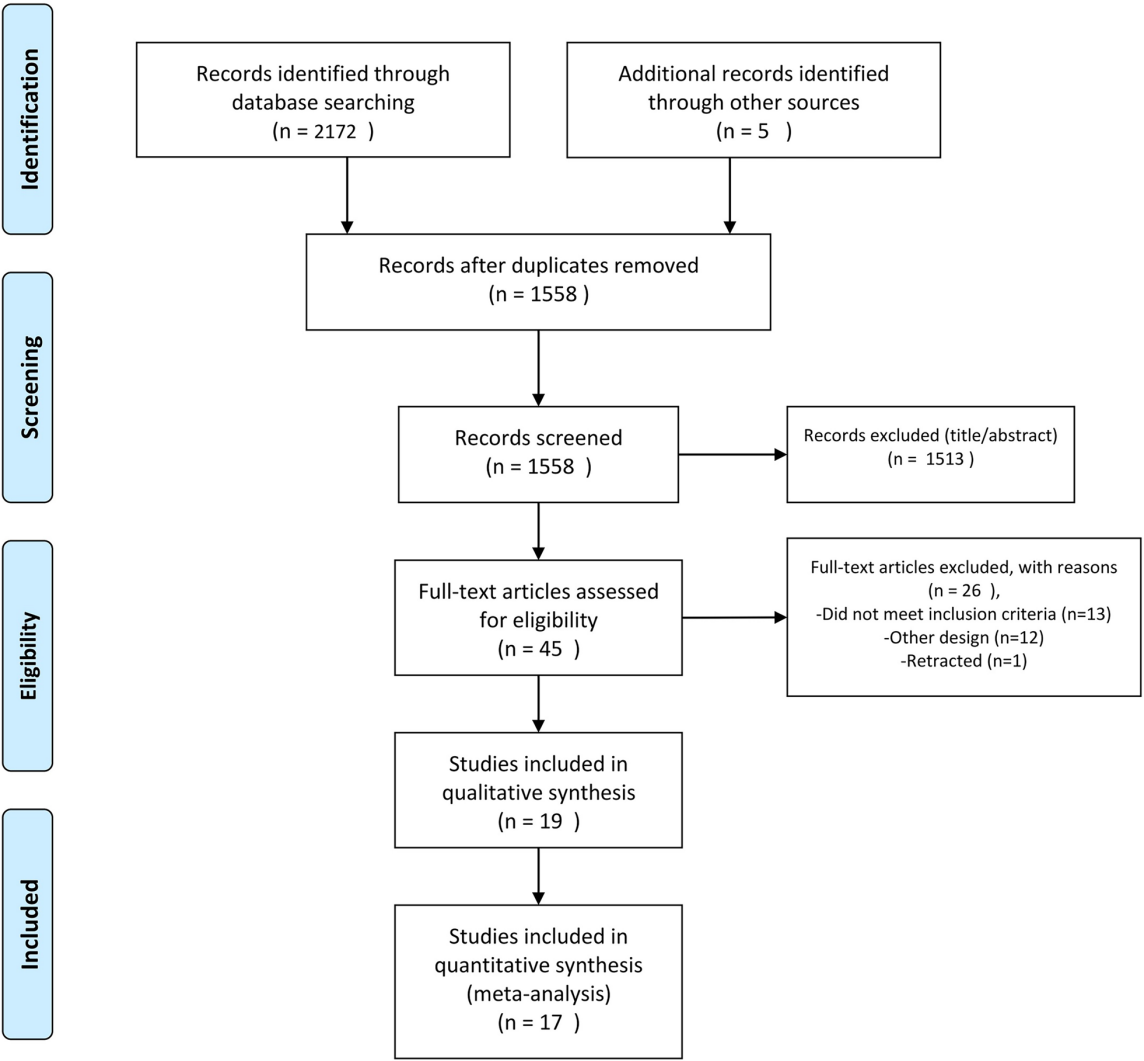
Evidence search and selection based on the PRISMA (Preferred reporting items for systematic reviews and meta-analyses) approach

We identified 2177 relevant studies through database searching and 619 duplicates were removed (Fig. 1). After screening the titles and abstract, 45 full-texts were reviewed and 19 RCTs were included. Due to lack of data of one of the studies, we attempted to contact the corresponding, but received no response [32]. Finally, a total of 19 and 17 studies (involving 4328 participants) were included in the systematic review and meta-analysis, respectively.

### Characteristics of included studies

Among the included studies, eleven were conducted in Asian countries, including India (n = 3), Iran (n = 2), Iraq (n = 1), Egypt (n = 1), Bangladesh (n = 2), Malaysia(n = 1), and Turkey (n = 1), five in the Americas, including Brazil (n = 2), Colombia (n = 1), Argentina (n = 2), two in Europe, including Spain (n = 1), Italy (n = 1), and one in Africa, including Nigeria (n = 1). The majority of participants were mild to moderate COVID-19 patients who had been diagnosed early. Some trials used a standard of care regimen in combination with ivermectin, although most of them did not specify the exact medications used. As these combinations may affect the efficacy of ivermectin, studies not specifying the SOC are probably biased. Detailed characteristics of each study is provided in Table 1.

**Table 1:** Summary characteristics of included randomized clinical trials evaluating the efficacy of ivermectin for patients with COVID-19

Author/Year	Country	Study type	Population	Intervention arm(s)	Control arm	Total (N)	Age	Funding	Standard of care	Quality	Ref
Pott-Junior /2021	Brazil	RCT	Mild COVID-19 patients	1-SOC + Ivermectin 100mcg/kg 2-SOC + Ivermectin 200mcg/kg 3-SOC + Ivermectin 400mcg/kg Exposure: N.A	SOC	32	Mean:49.4 (14.6)	Non-commercial phase 2a clinical trial conducted at the Federal University of S˜ao Carlos	N.A	High	[33]
Abd-Elsalam /2021	Egypt	RCT	Hospitalized mild to moderate COVID-19 patients	Single dose Ivermectin (12 mg) + SOC Exposure: For 3 days	SOC	164	Mean = 40.88	N.A	Paracetamol, oxygen, fluids (according to the condition of the patient), empiric antibiotic, oseltamivir if needed (75 mg/12 h for 5 days), and invasive mechanical ventilation with hydrocortisone for severe cases if PaO2 less than 60 mm Hg, O2 saturation less than 90% despite oxygen or noninvasive ventilation, progressive hypercapnia, respiratory acidosis (pH < 7.3), and progressive or refractory septic shock	High	[34]
Chaccour /2021	Spain	RCT	Non-severe COVID-19 patients and no risk factors	Single dose Ivermectin (400 μg/kg) Exposure: Single dose at the day of admission	Placebo	24	Range = 18–54, Median = 26	Barcelona Institute for Global Health and Clínica Universidad de Navarra	N.A	Low	[35]
Shahbaznejad /2021	Iran	RCT	Hospitalized COVID-19 patients	Single dose Ivermectin (0.2 mg/kg) + SOC Exposure: Single dose at the day of admission	SOC	69	Mean = 46.43(22.51)	N.A	Hydroxychloroquine and/or lopinavir/ritonavir	Low	[36]
Lopez-Medina /2021	Colombia	RCT	Mild COVID-19 patients	Single dose Ivermectin (300 μg /kg) Exposure: For 5 days	Placebo	398	Median = 37[26–45]	This study received an unrestricted grant from Centro de Estudios en Infectología Pediátrica (grant ScDi823)	N.A	High	[37]
Ravikirti /2021	India	RCT	Mild to moderate COVID-19 patients	Ivermectin 12 mg + SOC Exposure: For 2 days	Placebo + SOC	112	Mean = 52.5(14.7)	Financial disclosure provided	N.A	Low	[38]
Shakhsi Niaee /2021	Iran	RCT	Mild to severe COVID-19 hospitalized patients	1-Single dose ivermectin (200mcg/Kg) 2-Three low interval doses of ivermectin (200, 200, 200 mcg/Kg) 3-Single dose ivermectin (400mcg/Kg) 4- Three high interval doses of ivermectin (400, 200, 200 mcg/Kg) Exposure: Single dose at the day of admission	1-SOP 2- SOC + Placebo	180	Mean = 56	N.A	N.A	High	[39]
Babalola/2021	Nigeria	RCT	Confirmed COVID-19 patients	1-Ivermectin 6 mg 2-Ivermectin 12 mg Exposure: Twice a week for two weeks	Placebo + SOC	62	Mean = 44(14.7)	The Central Bank of Nigeria Health Sector Research and Development Intervention Scheme	lopinavir/ritonavir daily for 2 weeks plus	Low	[40]
Vallejos /2021	Argentina	RCT	Non-hospitalized COVID-19 patients	Ivermectin (24/36/48 mg based on patient weight) + SOC Exposure: For 2 days	Placebo + SOC	501	Mean = 42(15.5)	N.A	N.A	Low	[41]
Mohan /2021	India	RCT	Mild to moderate COVID-19 patients	1- Ivermectin 12 mg 2- Ivermectin 24 mg Exposure: Single dose at the day of admission	Placebo	125	Mean = 35.3(10.4)	Science and Engineering ResearchBoard, Department of Science and Technology, Government of India	N.A	Low	[42]
Okumus /2021	Turkey	RCT	Hospitalized COVID-19 patients	Ivermectin 200 mcg/kg/day Exposure: For 5 days	SOC	60	Mean = 62.20(13.00)	Afyonkarahisar Health Science University Scientific Research project Coordination Unit Project	N.A	High	[43]
Mahmud/2021	Bangladesh	RCT	Mild to moderate COVID-19 patients	Ivermectin 12 mg + doxycycline + SOC Exposure: Twice daily for 5 days	Placebo + SOC	400	Mean = 40(13.00)	N.A	Standard of care included administration of paracetamol, antihistamines, cough suppressants, vitamins, oxygen therapy according to indication and need, low molecular weight heparin according to indication, appropriate other broad-spectrum antibiotics, remdesivir injection, other antiviral drugs, and other drugs for associated comorbid conditions	Some concerns	[44]
Ahmed /2021	Bangladesh	RCT	Confirmed COVID-19 patients within 7 days of symptoms onset	1- Ivermectin 12 mg 2- Ivermectin 12 mg + doxycycline Exposure: For 5 days	Placebo	72	Mean: 42	This work was supported by Beximco Pharmaceutical Limited, Bangladesh	N.A	Some concerns	[45]
Krolewieckia /2021	Argentina	RCT	Confirmed COVID-19 patients within 5 days of symptoms onset	Ivermectin 0.6 mg/kg + SOC Exposure: For 5 days	SOC	45	N.A	This work was supported by grant IP-COVID-19–625	N.A	High	[32]
Saxena / 2021	India	RCT	Patients presenting with SARS-COV-2 infection	Ivermectin 0.2 mg/kg + SOC Exposure: Single dose at the day of admission	SOC	84	Median = 25 (16 – 32)	N.A	Symptomatic treatment, zinc, and vitamin C	High	[46]
Hashim /2021	Iraq	RCT	Outpatient/ Inpatient COVID-19 patients	Ivermectin 200 µg/kg per day for two days, and in some patients who needed more time to recover, a third dose 200 µg/kg PO per day was given 7 days after the first dose + Doxycycline + SOC Exposure: For 2/3 days	SOC	140	Mean = 48.7 ± 8.6	Alkarkh Health General Directorate	Vitamin C, D, and zinc, azithromycin, dexamethasone and oxygen supply if needed	High	[47]
Buonfrate /2022	Italy	RCT	Confirmed COVID-19 patients	1- Ivermectin 600 μg/kg, 2- Ivermectin 1200 μg/kg. (The authors claimed that in all RCTs to date, they have utilized the highest dose of Ivermectin) Exposure: For 5 days	Placebo	93	Median = 47.0 [31.0–58.0]	Italian Ministry of Health ‘Fondi Ricerca Corrente’ to IRCCS Sacro Cuore Don Calabria Hospital	N.A	Low	[20]
Lim / 2022	Malaysia	RCT	Confirmed COVID-19 patients within the first week of symptom onset	0.4 mg/kg body weight daily + SOC Exposure: For 5 days	SOC	490	Mean: 62.5(8.7)	N.A	Symptomatic therapy and monitoring for signs of early deterioration based on clinical findings, laboratory test results,and chest imaging	High	[19]
Reis / 2022	Brazil	RCT	Symptomatic SARS-CoV-2–positive adults	0.4 mg/kg body weight daily + SOC Exposure: For 3 days	SOC	1358	Median = 49.0 [38.0–57.0]	Funded by FastGrants and the Rainwater Charitable Foundation; TOGETHER ClinicalTrials.gov	Usual standard care for Covid-19 provided by health care professionals in Brazil	Low	[24]

### Risk of bias

**Fig. 2:**
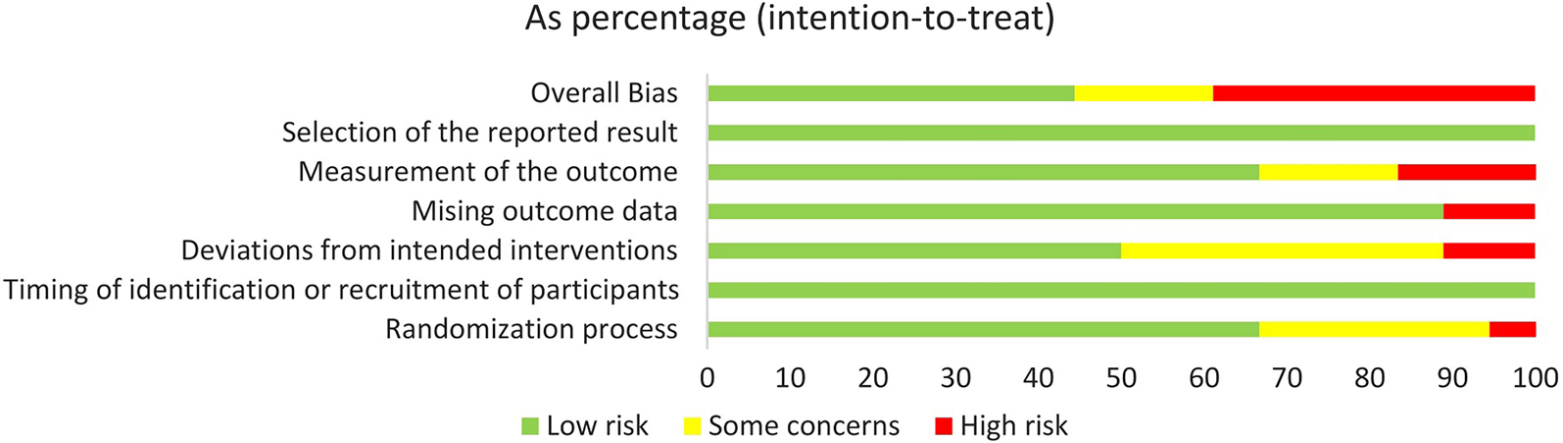
Overall risk of bias of included studies

Figure 2 shows the overall risk of bias of included studies. Risk of bias was rated as low in 7 trials, some concerns in 2 trials, and high in 9 trials. We have provided our detailed explanation for each study in the supplementary data (Additional file 1: Fig. S1).

### Primary outcomes

#### Mortality

**Fig. 3:**
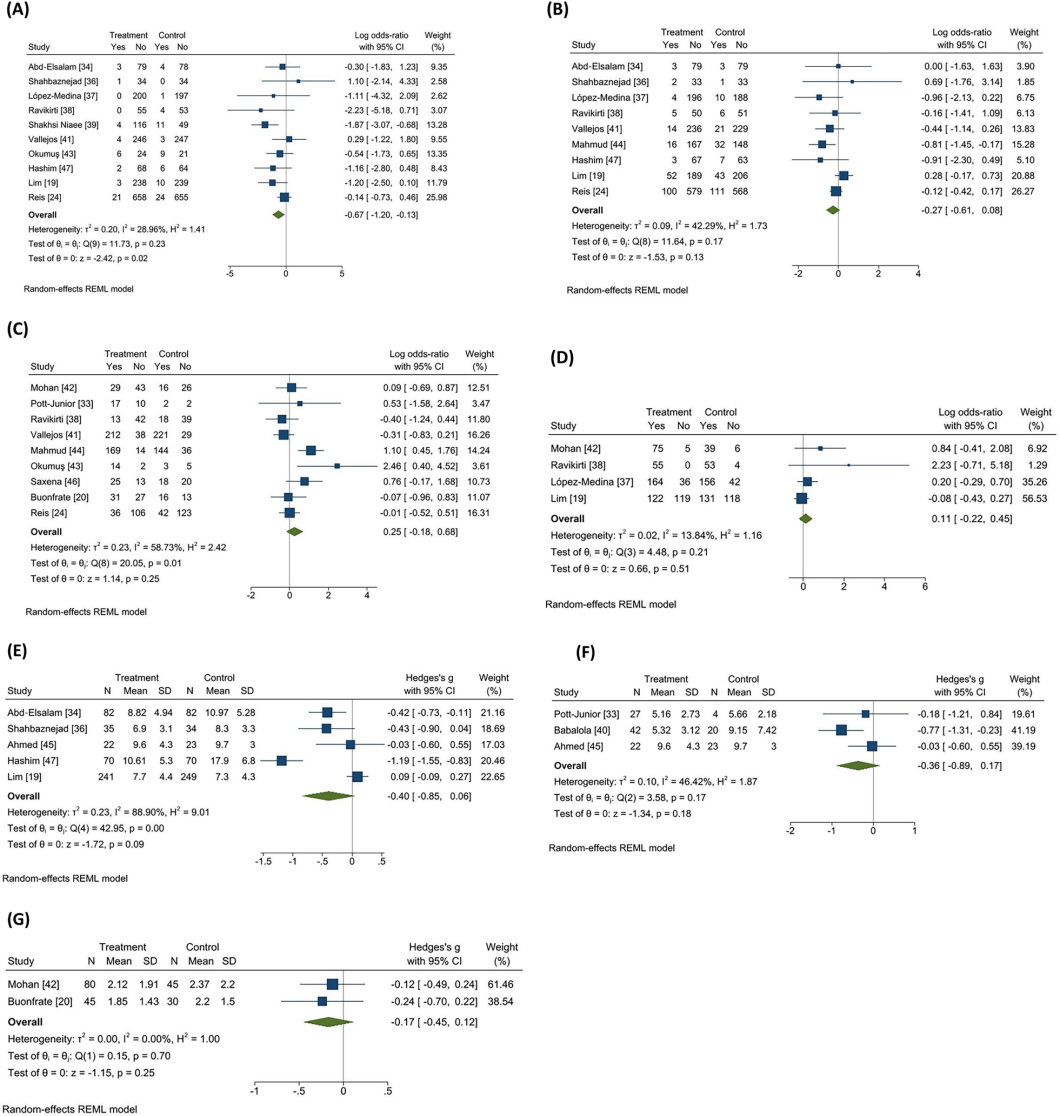
Forest plots showing the results of meta-analyses for primary outcomes. **A** Mortality, **B** Progression to severe disease, **C** Negative RT-PCR. Forest plots showing the results of meta-analyses for secondary outcomes. **D** Recovery, **E** Duration of hospitalization, **F** Time to negative RT-PCR, and **G** Viral load

**Fig. 4:**
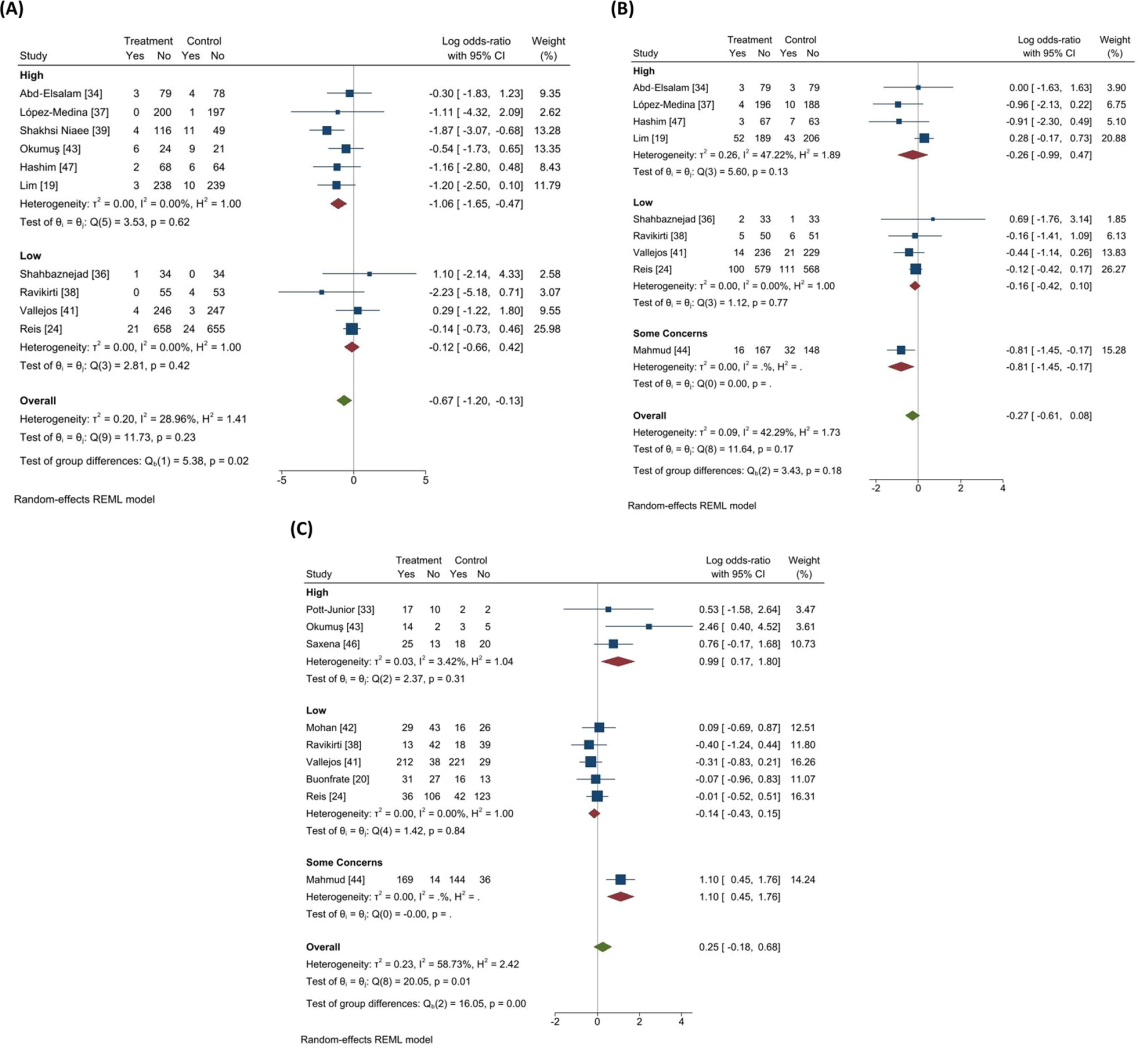
Meta-analysis of primary outcomes in subgroups based on the quality of included RCTs. In contrast to low biased trials, studies with a high/moderate risk of bias have indicated ivermectin efficacy in the majority of cases.** A** Mortality,** B** Progression to severe disease,** C** Negative RT-PCR

**Fig. 5:**
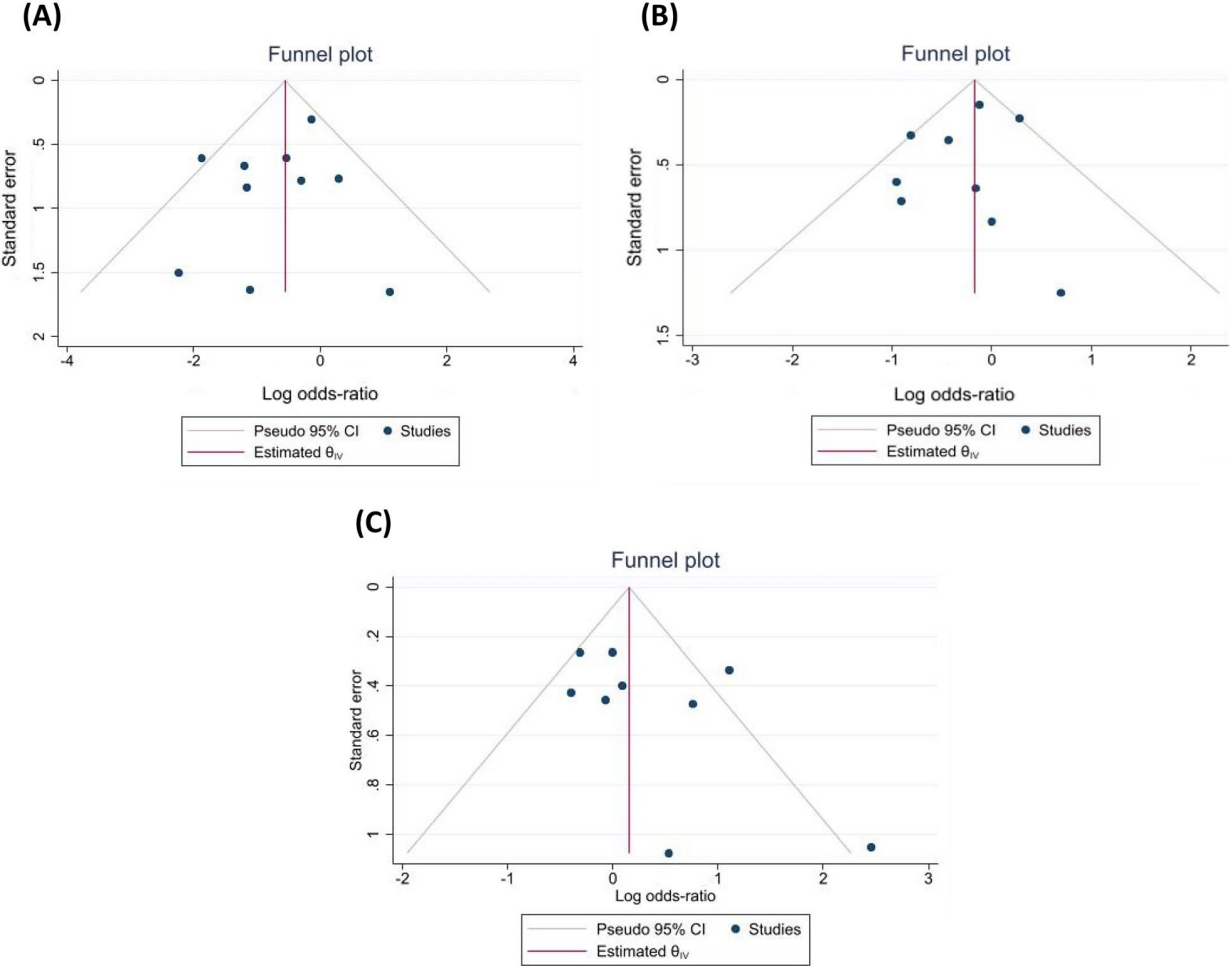
Funnel plot of primary outcomes for evaluation of publication bias. Secondary outcomes were not analyzed because of low number of studies included. **A** Mortality,** B** Progression to severe disease,** C** Negative RT-PCR

A total of 10 RCTs, including 3472 COVID-19 patients, reported a rate of mortality in their studies. Figure 3A) [19, 24, 34, 36–39, 41, 43, 47]. The pooled log OR was − 0.67 (95% CI − 1.20 to − 0.13) with low heterogeneity (I^2 = 28.96%, Tau = 0.20). The pooled results showed that ivermectin may have a possible effect on lowering the mortality rate. However, our subgroup analysis based on study quality found that ivermectin had no significant effect on mortality in trials with low ROB (log OR − 0.12, 95% CI 0.− 0.66–0.42; I2 = 00%, Tau = 0.00). While in trials with high ROB, there was a considerable reduction in mortality rate in the ivermectin group (log OR − 1.06, 95% CI − 1.65 to − 0.47; I2 = 0.00% Tau = 0.00) (Fig. 4A). To explore the possible effects of funding sources on the results of clinical trials, subgroup analysis between studies with and without funding was performed. There were no significant differences between funded (log OR − 0.44, 95% CI 0. − 1.00–0.12; I2 = 7.17%, Tau = 0.04) and non-funded (log OR − 0.71, 95% CI 0. − 1.65–0.22; I2 = 44.77%, Tau = 0.49) trials (Additional file 1: Fig. S2-A supplementary data). Sensitivity analysis using leave-one-out method revealed the overall effect was substantially altered when the study by Shakhsi Niaee et al. was omitted (Additional file 1: Fig. S3-A supplementary data) [39]. There was no publication bias based on inspection of funnel plot and Egger’s regression test (*p* = 0.65) (Fig. 5A).

#### Progression to severe disease

A total of 9 RCTs, including 3594 COVID-19 patients, reported progression to severe disease in their studies (Fig. 3B) [19, 24, 34, 36–38, 41, 44, 47]. The pooled log OR was − 0.27 (95% CI -0.61 to 0.08) with low heterogeneity (I^2 = 42.29%, Tau = 0.09). The pooled results showed that ivermectin does not have an effect on lowering the rate of progression to severe disease. Our subgroup analysis based on study quality revealed that ivermectin had no significant effect in disease progression in trials with low ROB (log OR -0.16, 95% CI -0.42 to 0.10; I2 = 0.00%, Tau = 0) and high ROB (log OR -0.26, 95% CI -0.99 to 0.47; I2 = 47.22%, Tau = 0.26) (Fig. 4B). There were no significant differences between funded (log OR − 0.29, 95% CI 0. −0.71 to − 0.13; I2 = 15.69%, Tau = 0.04) and non-funded (log OR -0.21, 95% CI 0. − 0.76–0.35; I2 = 53.77%, Tau = 0.19) trials (Additional file 1: Fig S2-B supplementary data). Sensitivity analysis using leave-one-out method revealed the overall effect was substantially altered by removing one study [19] (Additional file 1: Fig. S3-B supplementary data). There was no publication bias based on inspection of funnel plot and Egger’s regression test (*p* = 0.61) (Fig. 5B).

#### Negative RT-PCR

**Fig. 6:**
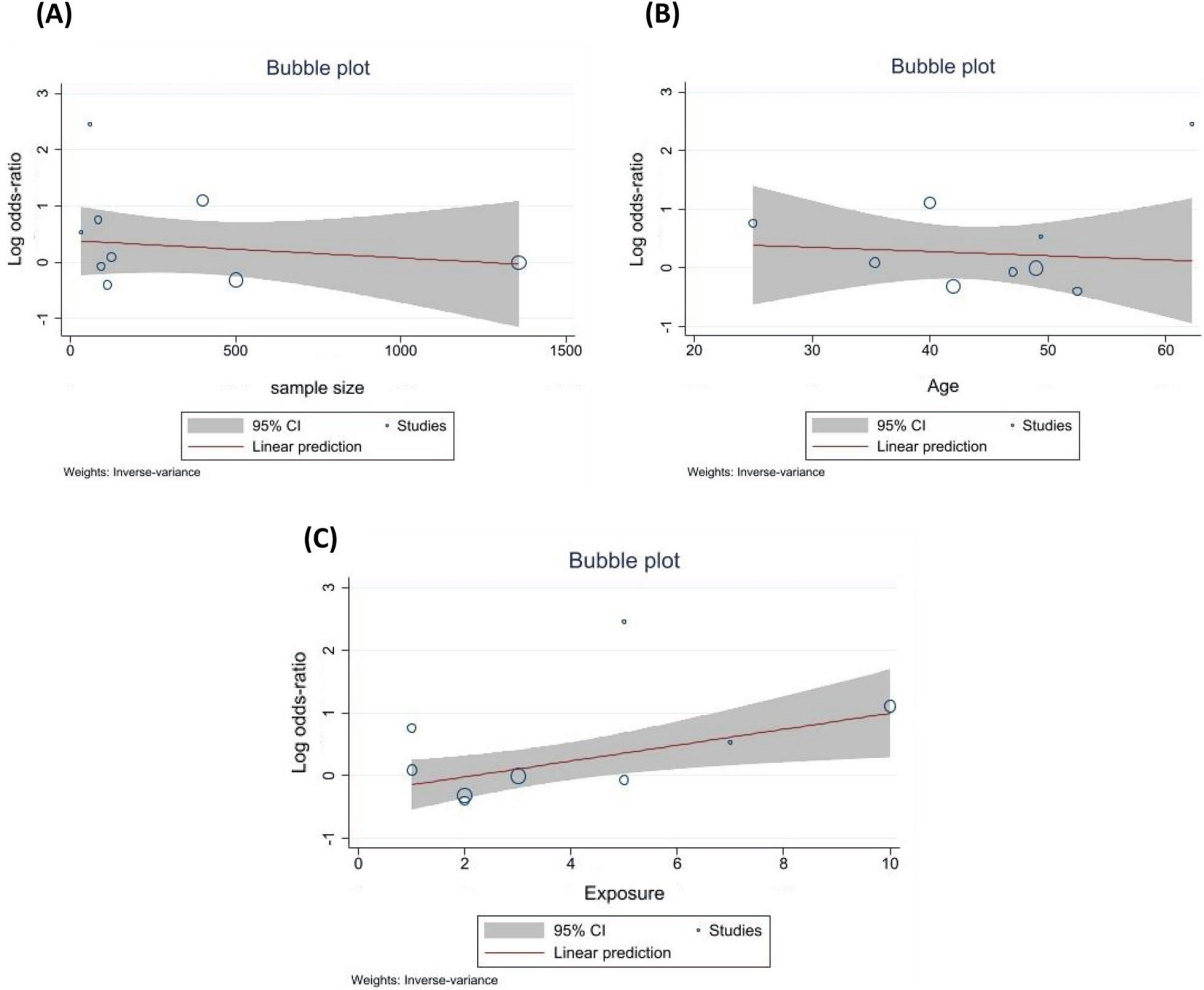
The correlation between negative RT-PCR rate and **A** sample size, **B** Age, and **C** exposure

A total of 9 RCTs, including 2679 COVID-19 patients, reported incidence of negative RT-PCR test in their studies (Fig. 3C) [24, 33, 38, 41–44, 46]. The pooled log OR was 0.25 (95% CI -0.18 to 0.68) with moderate heterogeneity (I^2 = 58.73%, Tau = 0.23). The pooled results showed that ivermectin does not have an effect on increasing the rate of negative RT-PCR. Our subgroup analysis based on study quality revealed that ivermectin had no significant effect in negative RT-PCR rate in trials with low ROB (log OR -0.14, 95% CI -0.43 to 0.15; I2 = 0.00%, Tau = 0). However, in trials with high ROB, there was a significant increase in negative RT-PCR rate in the ivermectin group (log OR 0.99, 95% CI 0.17–1.80; I2 = 3.42% Tau = 0.03) (Fig. 4C). No significant differences between funded (log OR 0.02, 95% CI 0  − 0.32 to 0.36; I2 = 0.00%, Tau = 0.00) and non-funded (log OR 0.48, 95% CI 0. -0.41 to 1.37; I2 = 80.46%, Tau = 0.49) studies were observed (Additional file 1: Fig S2-C supplementary data). Sensitivity analysis using leave-one-out method revealed the overall effect was not substantially altered when any single study omitted (Additional file 1: Fig. S3-C supplementary data). There was no publication bias based on visual inspection of funnel plot and Egger’s regression test (*p* = 0.10) (Fig. 5C). Although the heterogeneity was reduced by subgrouping studies based on their risk of bias, a meta-regression analysis was performed to assess other possible sources of heterogeneity using sample size, age, and exposure as covariates. The association between ivermectin and increased negative RT-PCR was significantly affected by drug exposure (*p* = 0.01). Furthermore, the results showed that the rate of negative RT-PCR was not influenced by sample size (*p* = 0.56) and age (*p* = 0.78) (Fig. 6).

### Secondary outcomes

#### Recovery

A total of 4 RCTs, including 1125 COVID-19 patients, reported an incidence of patients recovered in their studies (Fig. 3D) [19, 37, 38, 42]. The pooled log OR was 0.11 (95% CI − 0.22–0.45) with low heterogeneity (I^2 = 13.84%, Tau = 0.02). The pooled results showed that ivermectin does not have an effect on increasing the rate of recovery.

#### Duration of hospitalization

A total of 5 RCTs, including 908 COVID-19 patients, reported duration of hospitalization in their studies. (Fig. 3E) [19, 34, 36, 45, 47]. The pooled SMD was − 0.40 (95% CI − 0.85–0.06) with high heterogeneity (I^2 = 88.90%, Tau = 0.23). The pooled results showed that ivermectin does not have an effect on decreasing the duration of hospitalization.

#### Time to negative RT-PCR

A total of 3 RCTs, including 138 COVID-19 patients, reported time to negative RT-PCR in their studies. (Fig. 3F) [33, 40, 45]. The pooled SMD was − 0.36 (95% CI − 0.89–0.17) with low heterogeneity (I^2 = 46.2%, Tau = 0.10). The pooled results showed that ivermectin does not have an effect on decreasing the time to negative RT-PCR.

#### Viral load

A total of 2 RCTs, including 200 COVID-19 patients, reported viral load in their studies. (Fig. 3G) [20, 42]. The pooled SMD was − 0.17 (95% CI − 0.45–0.12) with low heterogeneity (I^2 = 0%, Tau = 0.00)**.** The pooled results showed that ivermectin does not have an effect on decreasing the viral load.

### Quality of evidence

The summary of findings and quality of evidence for study outcomes is available in Table 2.

**Table 2:** Ivermectin compared to control for COVID-19 patients

Patient or population: COVID-19 Intervention: Ivermectin Comparison: Standard of care or Placebo			
Outcomes	No of participants (studies) Follow up	Certainty of the evidence (GRADE)	Ref
Mortality	3472 (10 RCTs)	⨁◯◯◯ Very low^a,b^	[19, 24, 34, 36–39, 41, 43, 47]
Progression to severe or critical state	3594 (9 RCTs)	⨁⨁◯◯ Low^a,b^	[19, 24, 34, 36–38, 41, 44, 47]
Negative RT-PCR	2679 (9 RCTs)	⨁◯◯◯ Very low^a,b,c^	[24, 33, 38, 41–44, 46]
Recovery	1125 (4 RCTs)	⨁⨁◯◯ Low^a,b^	[19, 37, 38, 42]
Duration of hospitalization	908 (5 RCTs)	⨁◯◯◯ Very low^a,b,d^	[19, 34, 36, 45, 47]
Time to negative RT-PCR	138 (3 RCTs)	⨁⨁◯◯ Low^a,e^	[33, 40, 45]
Viral load	200 (2 RCTs)	⨁⨁◯◯ Low^b,e^	[20, 42]

*Explanations*

GRADE Working Group grades of evidence

*High certainty* We are very confident that the true effect lies close to that of the estimate of the effect

*Moderate certainty* We are moderately confident in the effect estimate: The true effect is likely to be close to the estimate of the effect, but there is a possibility that it is substantially different

*Low certainty* Our confidence in the effect estimate is limited: The true effect may be substantially different from the estimate of the effect

*Very low certainty* We have very little confidence in the effect estimate: The true effect is likely to be substantially different from the estimate of effect

^a^The proportion of information from studies at high risk of bias is sufficient to affect the interpretation of results. Crucial limitation for one criterion, or some limitations for multiple criteria, sufficient to lower confidence in the estimate of effect. Downgraded one or two levels based on the number of high-risk of bias studies

^b^Wide confidence interval (uncertainty about magnitude of effect). Downgraded one level

^c^Moderate heterogeneity. Downgraded one level

^d^High heterogeneity. Downgraded two level

^e^Small sample size. Downgraded one level

## Discussion

### Main findings

To our knowledge, this is the most comprehensive systematic review and meta-analysis of the efficacy of ivermectin among COVID-19 patients in peer-reviewed randomized clinical trials. Our aim was to appraise potential efficacy of ivermectin compared to placebo or SOC. Although our preliminary results suggest that ivermectin may reduce mortality, it is crucial to highlight that when trials with a high risk of bias are excluded, ivermectin results in a non-significant decrease in mortality. We found that in comparison with placebo or SOC, using ivermectin did not significantly change our outcomes, including progression to severe disease, negative RT-PCR, recovery, duration of hospitalization, time to negative RT-PCR, and viral load. Although no risk of publication bias was observed in primary outcomes, it is noteworthy that, based on the Cochrane handbook's suggestions, the power of these tests is too low for less than 10 studies to be included. We also identified that some registered RCTs finished without reporting results and so there may be publication bias. The overall certainty of evidence suggest that more research is needed and final conclusions should not be drawn based on the current findings and there is insufficient evidence to recommend it for therapeutic purposes in the context of health care at this time. To determine the impact of individual studies on the pooled estimate, a leave-one-out analysis was performed on primary outcomes. It was observed that omitting one study (Lim et al. [19]) considerably changed the pooled estimate of progression to severe disease. Therefore, further investigation into the results of this recently published study is necessary. Among 19 randomized trials included; 10 studies concluded that ivermectin may have a possible effect in treating COVID-19. To address the conflicts and clear up the gray area; we precisely assessed the risk of bias of studies using the Cochrane risk-of-bias tool for randomized trials. Of all included studies in our review, 9 had a high risk of bias, and 2 had moderate risk of bias. This level of bias raises concerns regarding the accuracy of the results and quality of the studies and should be noted to prevent wrong conclusions. Any decision made upon the findings of these studies should be carefully appraised before taking it into action to avoid possible complications. A study by Shakhsi-Niaee et al. [39] declared a significant reduction in mortality rate among ivermectin groups. Howerever, we do not consider it as a valid source of information due to the low quality of evidence and unclear or high risk of bias in multiple domains [15, 48, 49]. In addition, major sources of concern have been raised about this study [39], which may lead to retraction of this study in the future [15, 50].

### Findings of other meta-analyses

The first systematic review published on this topic by Padhy et al.[51] claimed a significant reduction in all-cause mortality and significant clinical improvement although stating a high risk of bias for all studies and very-low quality of evidence. Cruciani et al.[52] published a systematic review with the primary outcome of overall mortality and progression to severe disease including 11 RCTs with 2436 participants. They concluded that when the analysis was limited to patients with baseline severe disease, ivermectin significantly decreased mortality compared to mild to moderate disease. However, they stated that the quality of evidence was very low due to the risk of bias. Kow et al. [53] concluded a preliminary positive effect on reduction of all-cause mortality by reviewing 6 studies, although they declared a high risk of bias for 4 out 6 studies. Roman et al. [54] published a systematic review and meta-analysis with primary outcomes such as all-cause mortality, length of hospital stay, and AEs and secondary outcomes of SARS-CoV-2 clearance on respiratory samples, clinical improvement, need for mechanical ventilation, and severe AEs. Their quality assessment demonstrated a high risk of bias among 8 out of 10 studies, and they reported a low or very low quality of evidence for all outcomes. They found that ivermectin did not reduce primary or secondary outcomes in patients with mostly mild disease and claimed that ivermectin is not a viable option for treating COVID-19 and should be used only in the context of clinical trials. Deng et al. [55] published a systematic review which excluded retracted studies and articles withdrawn from preprint servers. They concluded that based on available evidence, ivermectin does not significantly alter the outcomes of the disease including viral clearance, duration of hospitalization, mortality and incidence of mechanical ventilation. Except for mortality, their findings were consistent with our study in terms of the efficaciousness of ivermectin in the setting of COVID-19. The authors of a retracted meta-analysis [23] recently provided an update on their prior conclusions, which were based on separating the included studies regarding the quality of the trials as determined by the Cochrane Risk of Bias tool. The authors included 12 trials (including Elgazzar et al.'s retracted study) to investigate the possible role of study quality in pooled results. Their findings are in line with our study, which demonstrates that studies with a high risk of bias or probable medical fraud were necessary to find a substantial positive effect of ivermectin on survival [56].

### Strengths and limitations

This review has strengths in several aspects. Our systematic search protocol was designed for obtaining comprehensive and up-to-date results from 6 databases. That helps to achieve a more accurate estimation of the effect of ivermectin. All of the studies included in our review are peer-reviewed RCTs meaning that we reviewed the highest level of evidence available to avoid deviation from the mainstream of evidence. We observed any supplementary data of the studies to maximize the amount of analyzed data and minimize errors. By excluding studies with questionable methodologies and inadequate follow-up periods, we avoided potential partiality. The evaluation of each outcome was based on the information obtained from at least 2 studies, and the certainty of the evidence was assessed for all reported outcomes. We also faced some limitations. Most of the studies included a small number of participants and presented low to moderate symptoms. Therefore, the assessment of patients with moderate to severe disease has remained a question. Risk of bias was assessed high for several studies, and the quality of evidence for most of the outcomes is low, hence our concern about the applicability of results approving ivermectin efficiency and safety. The majority of the research available did not elaborately elucidate their methodologies. In addition, number of studies had the standard of care or other co-interventions plus ivermectin as intervention and different type of comparators as control. This may affect the accuracy of findings of studies as a proper comparison may not be possible under these circumstances. According to the limitations mentioned above, further research involving large-scale RCTs with a broad spectrum of disease severity and longer follow-up periods is warranted in order to provide adequate evidence regarding the safety and efficacy of ivermectin use for treatment of COVID-19.

### In quest of an effective drug during a pandemic

**Fig. 7:**
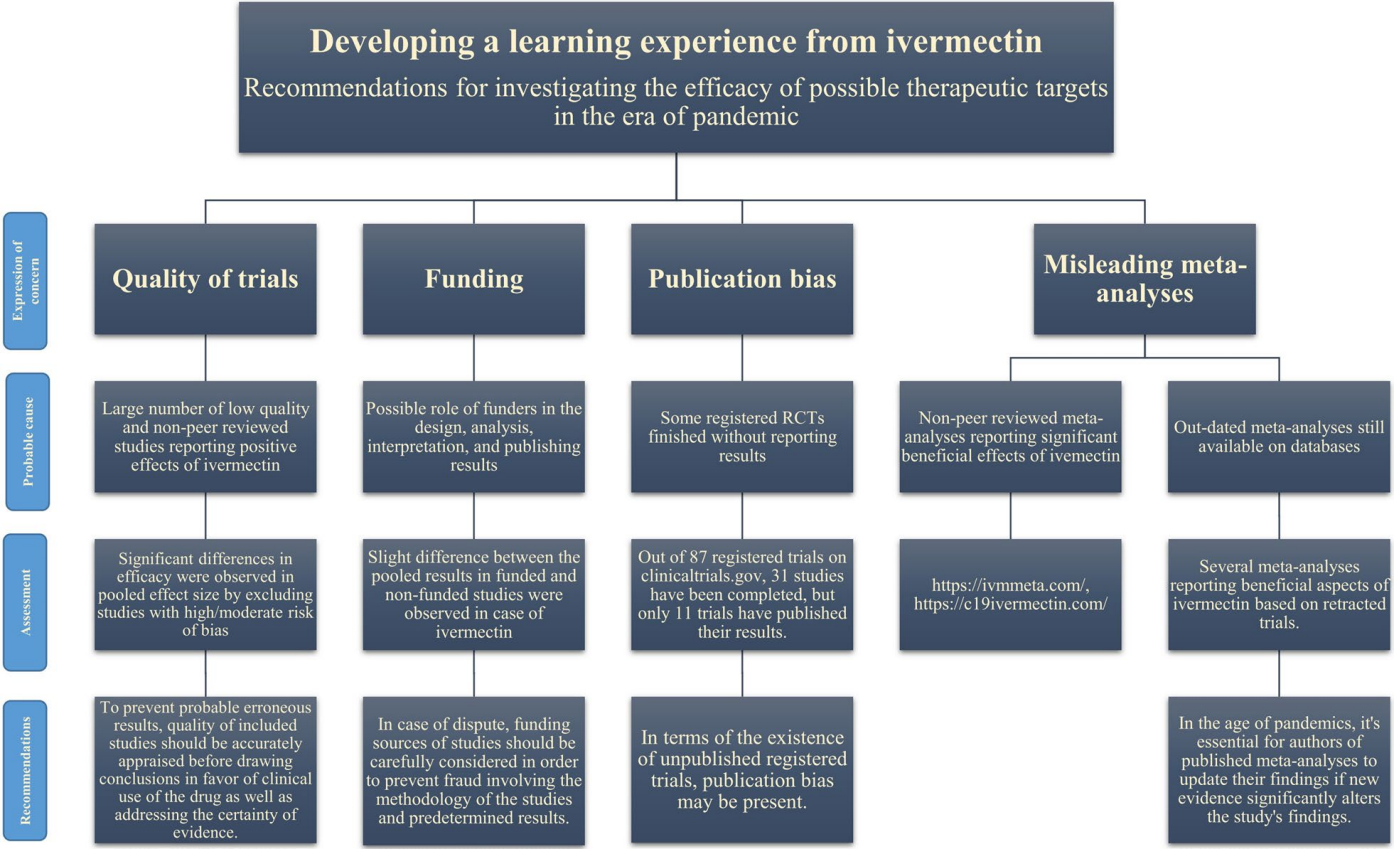
Recommendations for researchers and meta-analysis authors for assessing the efficacy of future proposed medications during a pandemic in light of what we've learnt from ivermectin

Recurrent surges in the mortality rates and economic consequences of the COVID-19 pandemic led to a distressing situation for societies. Accompanied by skepticism towards new drugs and vaccines and misinformation spread by media, this situation resulted in people making efforts in order to seek any accessible treatment regardless of whether health authorities approved it as a safe and efficacious treatment against the disease. One of these drugs was ivermectin, a well-known drug which has been widely used as an anti-parasitic drug for a long time. The idea of overcoming a pandemic with a previously used, widely available and low-cost drug made it challenging for scientists to prevent its public use. Cases were reported of individuals taking highly concentrated forms of this drug formulated for animals such as horses or prescriptions from physicians for treatment or prevention of COVID-19, resulting in hospitalization or ICU admission [57]. The lack of sufficient and concrete scientific evidence regarding the safety and efficacy of ivermectin was also a key factor in throwing the usage of this drug into question. Recent articles were arguing that studies evaluating the effect of ivermectin are biased and that there is a possibility of fraudulent manipulations in the methodology of RCTs [58, 59]. To overcome this predicament, it is of crucial importance to gather the already-existing evidence from relevant studies and meticulously evaluate the outcomes of this drug. Our study was designed to impartially address this issue to provide a clear perspective on the subject of ivermectin for clinicians and researchers. In the concept of COVID-19, useful lessons were learned from ivermectin for researching future potential therapeutic targets during a pandemic, which are critical for both researchers and clinicians. In addition, we identified four possible domains that must be evaluated by researchers whenever a new medication is proposed (Fig. 7).

The quality of included studies in meta-analyses affects the accuracy of the results. As there are controversies regarding ivermectin, we assessed the quality of studies to minimize the misconceptions. Many meta-analyses were conducted based on results obtained from retracted RCTs or those with high ROB without addressing their impact sufficiently. This leads to the spread of misinformation and the formation of low-quality evidence. The exclusion of studies with high or moderate risk of bias from our analyses on primary outcomes resulted in a significant difference in pooled effect results, suggesting that studies with high ROB play a major role in the current confusing state regarding the efficacy of this drug. To avoid this situation, the quality of included trials should be carefully evaluated before concluding that the drug is effective in pandemic situations.

To date, a number of systematic reviews have been published on this subject. However, most of them include studies by Elgazzar et al. [], or Samaha et al. [60] which now have been retracted from the databases or preprint studies with no peer-review process. Through our manual MEDLINE search, we identified several systematic reviews and meta-analyses based on the results of these fraught studies, reporting an independently significant effect of this drug in their analyses which results in confusion in both clinicians and patients [16–18, 61–64]. We highly advise systematic review and meta-analyses authors to keep themselves informed even after their work has been published in order to keep their findings updated and avoid producing misleading information. This is particularly noticeable in the context of an outbreak such as COVID-19, where there is a pressing need for demonstrating whether novel therapy options are clinically beneficial, and a plethora of low-quality and dubious research are widely available due to the pressing demand.

There have been several websites providing real time meta-analyses of ivermectin studies (https://ivmmeta.com/#top, https://c19ivermectin.com/) reporting its significant beneficial effect based on non-peer-reviewed low quality trials. They also lack protocol registration including methodologies, search strategies, inclusion criteria, quality assessment of the included studies, and the certainty of the evidence of the pooled estimates [14]. This is significant since the majority of individuals can find these websites by searching "ivermectin meta-analysis," which might cause misunderstandings.

Funding source of the studies and its association with the reported results raises concerns regarding the validity of results of the studies since conflicts of interest may affect the outcome of the trials. There are some studies suggesting significant association between the source of funding and outcome of the studies [65, 66]. In these cases, researches may be subject to methodological bias in favor of effectiveness of the intervention, reporting positive effects more frequently. Therefore, it is of great importance to ensure that the results of the study are not influenced by the funding source, and the objectivity of the study is preserved. [67] We performed a subgroup analysis comparing studies that provided information regarding their funding source with studies that did not mention theirs. For most of the outcomes, there was a non-significant difference between subgroups in terms of reporting the efficacy of ivermectin, however, a slight difference was observed in mortality rates. In such controversial state with possible conflicts of interest, it is crucial to carefully consider the funding source of the studies so as to reduce the probability of the study results to be affected by such confounding factors. This assurance could be achieved by journals and editors emphasizing the importance of this issue and demanding authors to provide sufficient information about their funding.

There are some concerns regarding the misprescription of this drug that should be addressed [9, 10, 68]. Ivermectin is a currently used antiparasitic drug with proven efficacy against several diseases such as scabies and filariasis, thus shifting its use towards the treatment of COVID-19 will result in the diversion of limited health-care sources, leaving us deprived of supplies necessary for combating burden of tropical diseases including the two mentioned above [58]. Furthermore, the minimum concentration needed to obtain the anti-SARS-CoV-2 effect of ivermectin is 5 μM, considerably higher than 0.28 μM, the maximum plasma concentration achieved in vivo with a dose of approximately 1700 μg/kg (about nine times the dosificaition approved by FDA) [14]. In January 2022, a study by Buonfrate et al. investigated the efficacy and safety of high-dose ivermectin in the early treatment of COVID-19 patients [20]. The authors claimed that in all RCTs to date, they have utilized the highest dose of ivermectin in the concept of a clinical trial. Their treatment arm included ivermectin single dose 600 μg/kg, and 1200 μg/kg. However, they reported a non-significant difference between ivermectin and placebo in their primary outcome, which was viral reduction. It is noteworthy that they have also seen some adverse events in both the 600 g/kg and 1200 g/kg groups, such as photophobia, visual impairment, abdominal pain, nausea, and fatigue. This could suggest that the medicine is unsafe at higher doses.

## Conclusion

Our review showed that ivermectin does not have any significant effect on most outcomes such as progression to severe disease, negative RT-PCR, recovery, duration of hospitalization, time to negative RT-PCR, and viral load. It can possibly decrease mortality, however most of the supporting data are from highly biased studies. Due to the low certainty of evidence and large number of studies with high/moderate risk of bias, there is still a need for further investigation with larger sample sizes to show whether ivermectin is a choice in the setting of COVID-19 patients with more confidence. In order to maintain our results up to date, we will prepare a major update to our work if new evidence significantly affects the study findings.

## Supplementary Information

**Additional file1** **Table S1:** Databases searched and search strategies employed. **Fig S1:** Quality assessment based on ROB2 checklist. **Fig S2:** Subgroup analysis (funding) (random-effects model). **Fig S3:** Sensitivity analyses of primary outcomes based on leave-one-out method.

## Data Availability

Data sharing is available by contacting corresponding author.
