# Supplementary material for: Ivermectin under scrutiny: a systematic review and meta-analysis of efficacy and possible sources of controversies in COVID-19 patients
Source: Virol J. 2022 Jun 13;19:102. doi: 10.1186/s12985-022-01829-8 (PMC9191543; doi:10.1186/s12985-022-01829-8)
Supplement: Supplementary file 1 — Additional file1 Table S1: Databases searched and search strategies employed. Fig S1: Quality assessment based on ROB2 checklist. Fig S2: Subgroup analysis (funding) (random-effects model). Fig S3: Sensitivity analyses of primary outcomes based on leave-one-out method. [file 12985_2022_1829_MOESM1_ESM.docx]

**Supplementary data**

**Table S1: Databases searched and search strategies employed**

| **Database** | **Search strategy** |  |
| --- | --- | --- |
| PubMed | (((((("COVID-19"[Mesh]) OR ("SARS-CoV-2"[Mesh])) OR (COVID-19)) OR (Coronavirus)) OR (nCoV)) OR (SARS-Cov-2)) AND ((((((("Ivermectin"[Mesh]) OR (MK-933)) OR (Stromectol)) OR (Mectizan)) OR (Eqvalan)) OR (Ivomec)) OR (Ivermectin)) |  |
| Web of Science | **(TOPIC:** (COVID19) OR **TOPIC:** (Coronavirus) OR **TOPIC:** (nCoV) OR **TOPIC:** (SARS-CoV-2) OR **TOPIC:** (COVID), Indexes=SCI-EXPANDED, SSCI, A&HCI, ESCI Timespan=All years**) AND (TOPIC:** (Ivermectin) OR **TOPIC:** (MK-933) OR **TOPIC:** (Stromectol) OR **TOPIC:** (Mectizan) OR **TOPIC:** (Eqvalan) OR **TOPIC:** (Ivomec), Indexes=SCI-EXPANDED, SSCI, A&HCI, ESCI Timespan=All years**)** |  |
| Scopus | ( ( TITLE-ABS-KEY ( covid-19 ) OR TITLE-ABS-KEY ( coronavirus ) OR TITLE-ABS-KEY ( ncov ) OR TITLE-ABS-KEY ( sars-cov-2 ) OR TITLE-ABS-KEY ( covid ) ) ) AND ( ( TITLE-ABS-KEY ( ivermectin ) OR TITLE-ABS-KEY ( mk-933 ) OR TITLE-ABS-KEY ( stromectol ) OR TITLE-ABS-KEY ( mectizan ) OR TITLE-ABS-KEY ( eqvalan ) OR TITLE-ABS-KEY ( ivomec ) ) ) |  |
| Cochrane library | ((Covid):ti,ab,kw OR (COVID-19):ti,ab,kw OR (Coronavirus):ti,ab,kw OR (nCoV):ti,ab,kw OR (SARS-CoV-2):ti,ab,kw) AND ((Ivermectin):ti,ab,kw OR (MK-933):ti,ab,kw OR (Stromectol):ti,ab,kw OR (Mectizan):ti,ab,kw OR (Eqvalan):ti,ab,kw OR (Ivomec):ti,ab,kw) |  |
| Google scholar, Clinicaltrials.gov | allintitle: COVID ivermectin OR allintitle: Coronavirus ivermectin OR allintitle: SARS-Cov-2 ivermectin OR allintitle: COVID-19 ivermectin OR allintitle: nCOV ivermectin  allintitle: COVID MK-933 OR allintitle: Coronavirus MK-933 OR allintitle: SARS-Cov-2 MK-933  OR allintitle: COVID-19 MK-933 OR allintitle: nCOV MK-933  allintitle: COVID Stromectol OR allintitle: Coronavirus Stromectol OR allintitle: SARS-Cov-2 Stromectol OR allintitle: COVID-19 Stromectol OR allintitle: nCOV Stromectol  allintitle: COVID Mectizan OR allintitle: Coronavirus Mectizan OR allintitle: SARS-Cov-2 Mectizan OR allintitle: COVID-19 Mectizan OR allintitle: nCOV Mectizan  allintitle: COVID Eqvalan OR allintitle: Coronavirus Eqvalan OR allintitle: SARS-Cov-2 Eqvalan OR allintitle: COVID-19 Eqvalan OR allintitle: nCOV Eqvalan  allintitle: COVID Ivomec OR allintitle: Coronavirus Ivomec OR allintitle: SARS-Cov-2 Ivomec OR allintitle: COVID-19 Ivomec OR allintitle: nCOV Ivomec |  |

**Fig S1: Quality assessment based on ROB2 checklist**


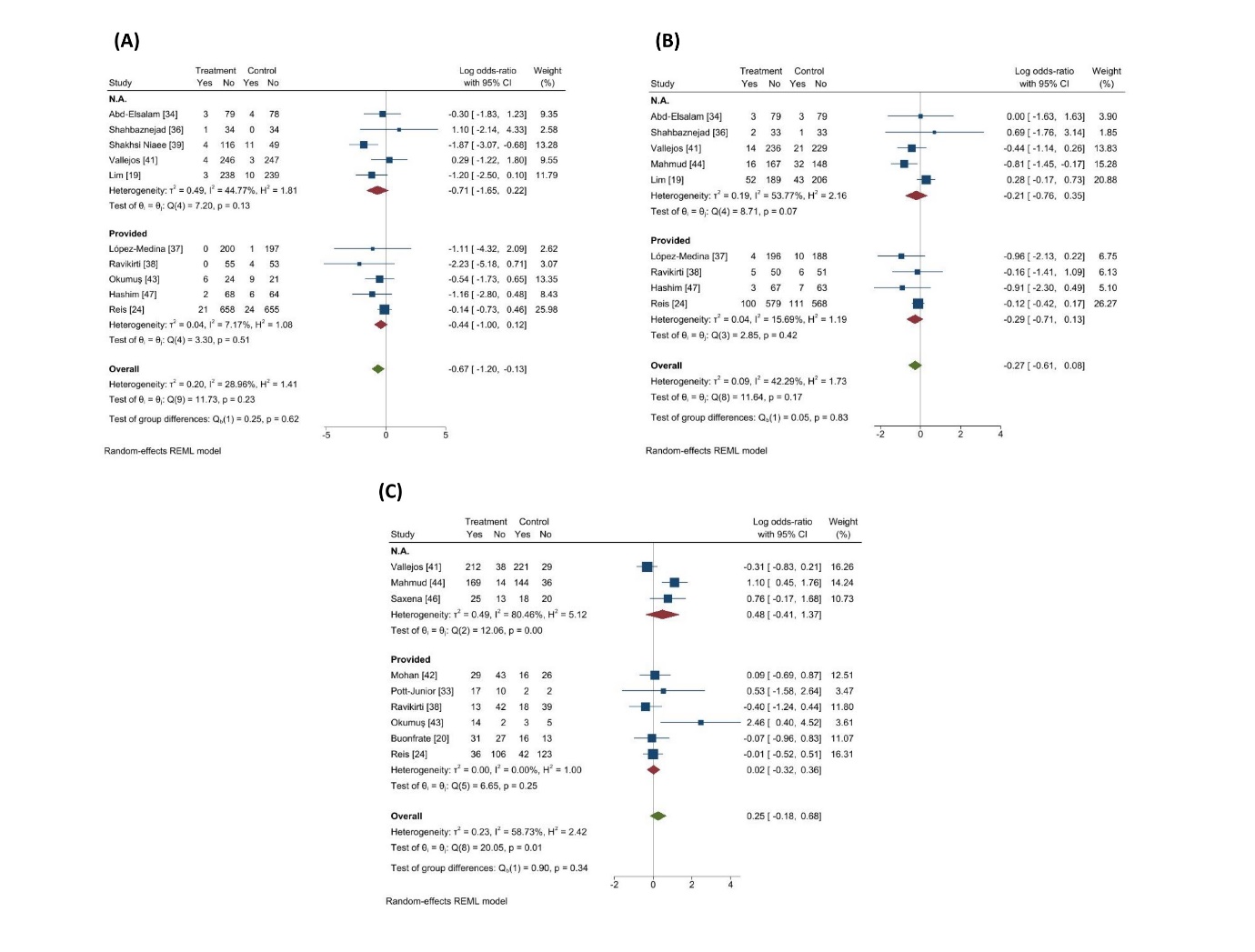


**Fig S2: Subgroup analysis (funding) (random-effects model)**


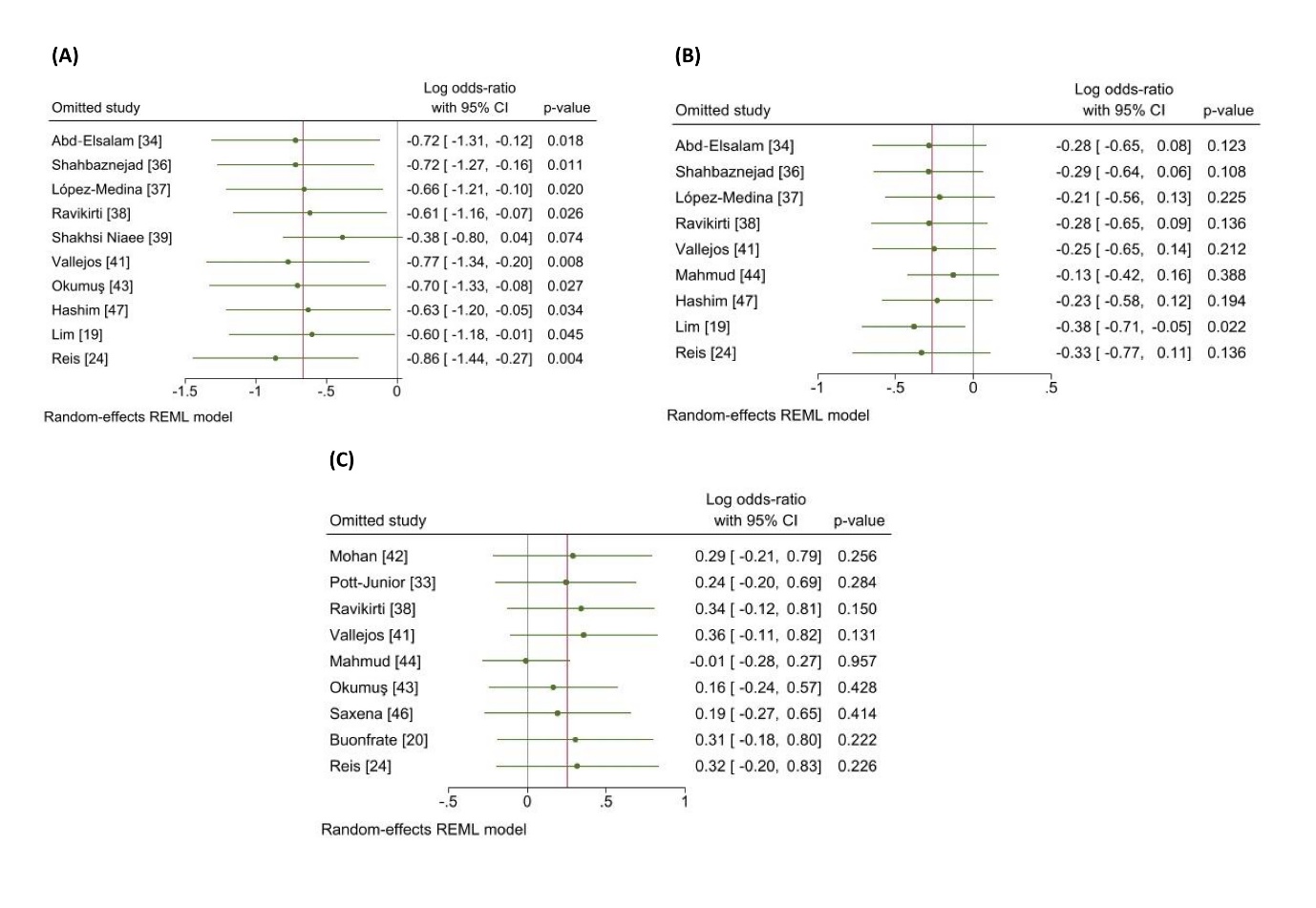


**Fig S3: Sensitivity analyses of primary outcomes based on leave-one-out method.**
